# Nothing but lung and bones: Longitudinal evolution and quantitative analysis in a case of idiopathic diffuse pulmonary ossification

**DOI:** 10.1016/j.radcr.2022.01.059

**Published:** 2022-02-20

**Authors:** Aldo Carnevale, Serena Chiarello, Giovanni Lanza, Alberto Cossu, Melchiore Giganti, Brunilda Marku

**Affiliations:** a Radiology Unit, Department of Translational Medicine, University of Ferrara, Ferrara, Italy; b Pathology Unit, Department of Translational Medicine, University of Ferrara, Ferrara, Italy; c Radiology Unit, Azienda Ospedaliero-Universitaria di Ferrara, Ferrara, Italy; d Radiology Unit Department of Translational Medicine, University of Ferrara, Ferrara, Italy; e Research Centre on Asthma and COPD, Department of Medical Sciences, University of Ferrara, Ferrara, Italy

**Keywords:** Diffuse lung disease, Diffuse pulmonary ossification, Quantitative analysis

## Abstract

A 77-year-old Caucasian man, a former surveyor in a chemical company, underwent a chest X-ray (CXR) as a follow-up exam for a melanoma of the back, surgically removed.

CXR showed interstitial thickening in both lower lobes; then, a high-resolution computed tomography of the chest (HRCT) was performed to further investigate these findings, revealing multiple small, calcified nodules with branching appearance at both lung bases.

Clinical examination and exposure history were negative, except for a decrease in diffusing capacity for carbon monoxide resulting from pulmonary function tests.

Surgical lung biopsy was performed; histology revealed numerous nodules and branching tubules of bone tissue, some of which with marrow elements.

After multidisciplinary discussion of the case, a diagnosis of idiopathic diffuse pulmonary ossification (DPO) was considered.

Clinical status of the patient was stable over time, despite the increase in extent of calcifications.

DPO is an uncommon condition that should be considered in different clinical-radiological settings; multidisciplinary discussion is essential for the final diagnosis.

## Introduction

Diffuse pulmonary ossification (DPO) is an unusual condition characterized by metaplastic ossification of the lung parenchyma.

It has been described in diverse contexts, even in patients with normal lungs (idiopathic DPO); however, it usually occurs against a background of pre-existing pulmonary, cardiac or metabolic disorders [2,3].

DPO has no specific clinical features and is usually an incidental finding during surgical lung biopsy or autopsy.

Two different subtypes of DPO have been recognized based on their histopathological pattern, namely nodular and dendriform [5,6]. The nodular type presents with rounded, calcified nodules within alveolar spaces and is more often encountered in patients with pulmonary congestion due to heart failure or mitral valve disease. Instead, the dendriform variant is characterized by branching calcifications arising from interstitial septa, usually containing marrow elements, and is more frequently associated with lung fibrosis.

The pathogenesis of DPO remains unclear; however, both external triggers and genetic predisposition may be involved [7]. This abnormality is most likely due to the precipitation of calcium and phosphate in lung tissue consequent to hypercalcemia, but it may also be interpreted as a metaplastic response to chronic lung injury by pulmonary fibroblasts and macrophages which can differentiate into osteoblasts in response to local hypoxia or acidosis [8]. Some authors have reported that DPO could be related to low-level, chronic gastric acid aspiration [8].

Patients with idiopathic DPO may remain asymptomatic for many years but do usually develop respiratory symptoms between the third and fourth decades, typically non-productive cough, dyspnea, chest pain, and asthenia. In some cases, it can progress into lung fibrosis, respiratory or cardiac failure [6,9].

We presented a case of idiopathic DPO diagnosed in an asymptomatic patient; we sought to determine the longitudinal evolution of the condition, also applying computer-aided analysis for calcium content quantification, and we showed a substantial disconnection between symptoms and radiological progression over time.

## Case report

A 77-year-old retired Caucasian man, a former surveyor in a chemical company, underwent a chest X-ray (CXR) in 2015 as part of surveillance for stage II melanoma of the back detected in 2006 and surgically removed.

**Fig. 1:**
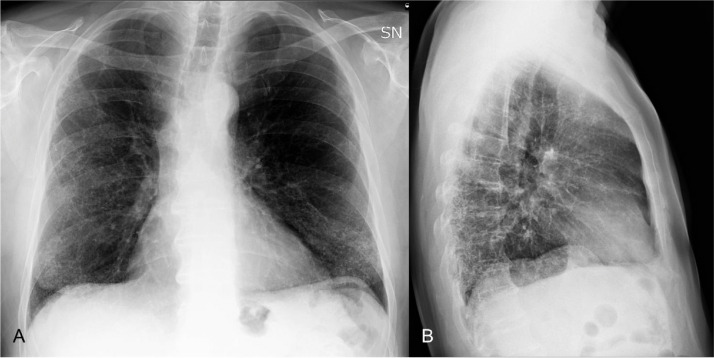
A - B. Chest radiograph, posterior-anterior and lateral view, demonstrating reticulo-nodular pattern involving above all the lower lobes.

CXR showed bilateral, interstitial thickening with reticular-nodular pattern mainly involving the lower lobes (Fig. 1).

**Fig. 2:**
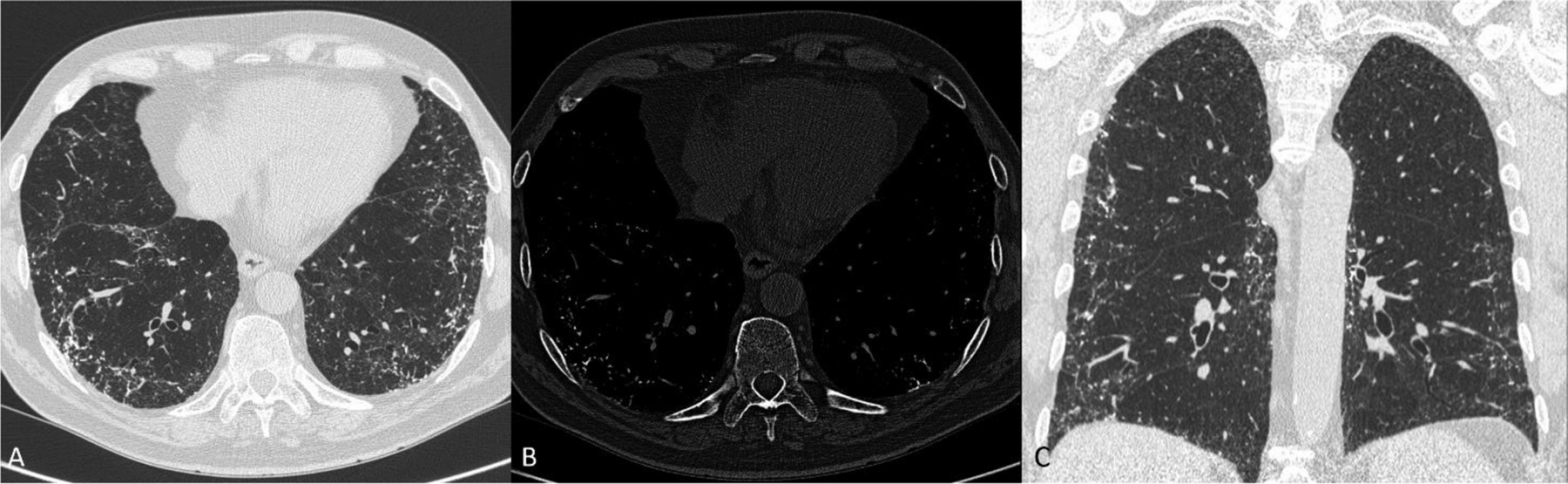
A - B - C. High resolution computed tomography (HRCT) scan, lung (A) and bone window (B), showing small, calcified nodules and branching opacities at lung bases, in absence of signs of lung fibrosis. Coronal reconstructed image depicts the cranio-caudal distribution of the abnormalities.

A high-resolution computed tomography of the chest (HRCT) was performed to further investigate these findings, revealing multiple, very small (<4 mm diameter) calcified nodular opacities with a dendriform, that is branching, appearance at both lung bases (Fig. 2A-C), absent in a previous CT scan performed for melanoma staging in 2006.

The patient was a former smoker (15 PY), with a medical history of hypertension and unstable angina since 1983. A detailed exposure history was negative. He did not report either drinking alcohol to excess or using recreational drugs. He was receiving therapy with P2Y12 inhibitor, diuretic, beta-blocker, calcium channel-blocker, angiotensin receptor-blocker, and statin.

Sporadic crackles at lung bases were reported during lung auscultation. Blood count, serum chemistry, autoantibodies and blood gas analysis were within normal limits, particularly the phosphocalcic values, except for elevated glycemia (120 mg/dl). Echocardiography was normal, whereas pulmonary function tests revealed an isolated decrease in diffusing capacity for carbon monoxide (DL_CO,_ 61% of predicted value).

Bronchoscopy with bronchoalveolar lavage (BAL) was consequently performed; results of cytologic and microbiologic analysis were normal (neutrophils 79%, lymphocytes 1%; macrophages 20%; total CD3+ 83%, CD3+ CD4+ 29%, CD3+ CD8+ 53%, CD16+ 12%, CD19+ 1%).

Salivary gland biopsy was obtained in order to exclude amyloidosis.

**Fig. 3:**
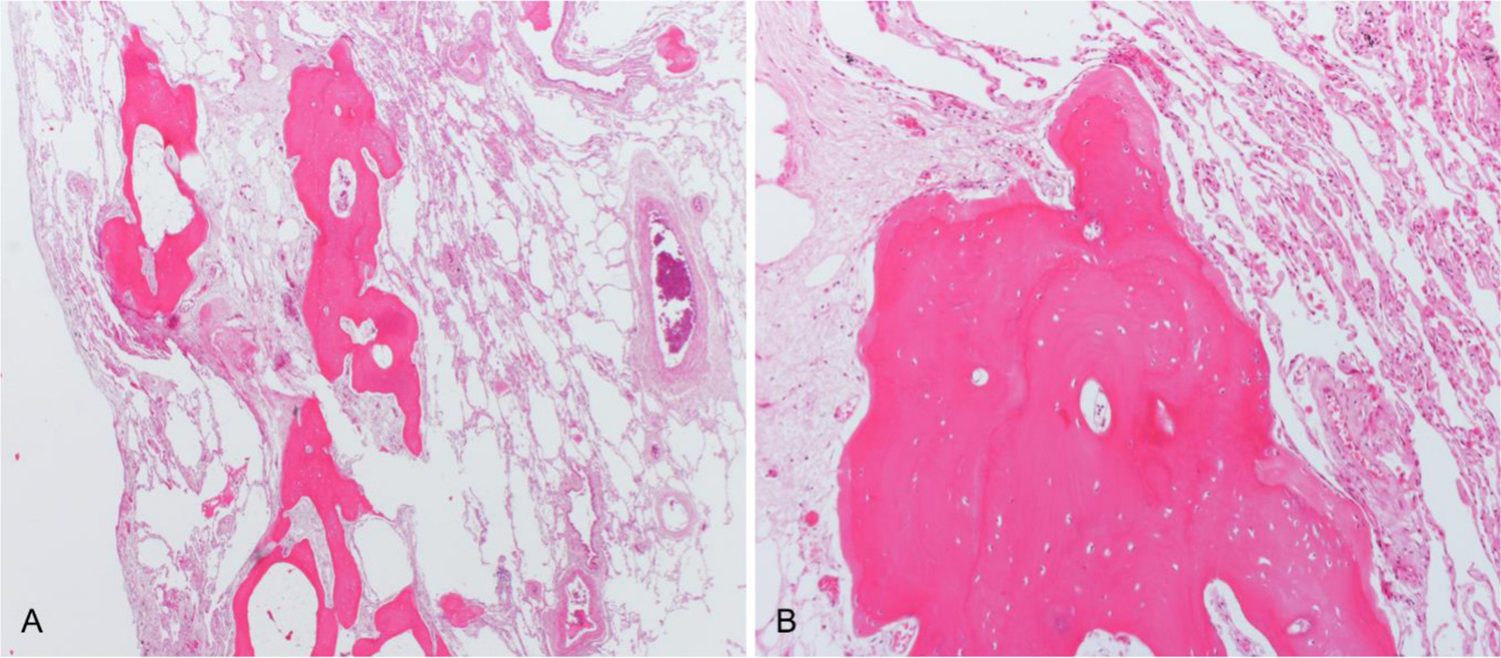
A - B. Lung biopsy demonstrating branching osseous structures within the interstitium, surrounded by mild fibrosis. Some of the bone nodules contain fat marrow.

The patient underwent surgical lung biopsy of the upper and lower right lobes. Histology revealed numerous nodules and branching tubules of bone tissue, with mature bone-marrow cells among bone spicules, in association with mild septal fibrosis and inflammatory infiltrates (Fig. 3 A, B).

In light of these findings, after multidisciplinary discussion of the case, a diagnosis of idiopathic diffuse pulmonary ossification (DPO) was considered.

Annual follow-up by means of pulmonary function tests and CXR was recommended.

**Fig. 4:**
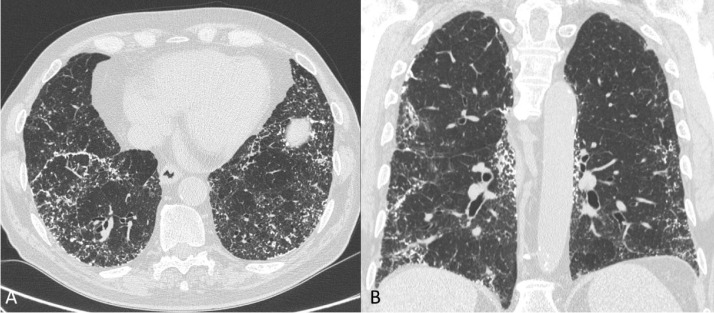
A - B. Follow-up HRCT showing an increase in extent of pulmonary calcifications (axial and coronal reconstructions in A and B, respectively).

A HRCT study performed in 2019, 4 years after the diagnosis, showed an increase in the extent of calcifications with no signs of lung fibrosis (Fig. 4 A, B).

A threshold-based method was adopted to quantify pulmonary calcium content at baseline and follow-up CT examinations using the calcium quantification tool integrated in the RIS/PACS system of the Radiology Department (Fenix Elco Health Systems/Carestream Health, Rochester, NY). Multiple regions of interest (ROIs) were manually drawn on the lung fields using mediastinum reconstructed series. We calculated a 633% increase in the volume of pulmonary calcifications, a monthly rate of change in the calcium content of the lungs of 417.5 mm^3^ and 87.5 mg, and a yearly change of 5010 mm^3^ and 1050.3 mg.

In spite of this, the patient's physical status was stable over time.

## Discussion

We report the case of a patient in whom idiopathic DPO was incidentally found, and the clinical and radiological evolution over time is described. Quantitative analysis was performed to estimate the increase in lung calcifications during follow-up.

Pulmonary calcifications are extremely frequent in chest imaging, and can be associated to a broad spectrum of conditions [1].

DPO is an unusual condition characterized by metaplastic ossification of the lung parenchyma. It has been described in diverse settings, but it usually occurs in a context of pre-existing pulmonary, cardiac or metabolic disorders [2,3], particularly pulmonary congestion secondary to mitral stenosis, after severe lung injury, or in association with fibrosing interstitial lung disease, the idiopathic variant being very rare (1.63 of 1000 cases at autopsy) [4]. Its pathogenesis is still unclear.

Chest CT has a high diagnostic yield for pulmonary ossification; however, descriptions of CT findings in patients who do not have underlying interstitial lung disease are limited. CT imaging is obviously more accurate than CXR, since it clearly depicts the density of the lung abnormalities and their exact distribution. At HRCT, DPO appears as bilateral small, calcified nodules, few millimeters in diameter, located in the peripheral interstitium and mainly involving the lung bases. Multiple contiguous nodules are composed of branching structures with a tree-like pattern [8].

The differential diagnosis for profuse calcified micronodules seen at chest CT is not wide. Indeed, pulmonary alveolar microlithiasis could theoretically represent a differential diagnosis for DPO. However, calcifications in this latter case are much smaller than in DPO, usually described as “sand-like,” and tend to accumulate along interlobular septa or bronchovascular bundles [3]. Small, calcified granulomata, for example associated to pulmonary tuberculosis and other granulomatous diseases, may be otherwise considered, however they tend to be more scattered, without the typical pattern of distribution observed in DPO.

Multidisciplinary discussion is often crucial for the management and final diagnosis of diffuse lung diseases [10]. In these settings, differential diagnosis should be made with other diseases producing lung calcifications (particularly, granulomatous infections, amyloidosis, pneumoconiosis, pulmonary alveolar microlithiasis, or metastatic calcifications) [1].

Cytologic and microbiologic tests on the BAL are often unremarkable in idiopathic DPO.

Histology represents the gold standard for diagnosis as it shows the bone metaplasy and the potential presence of fat or marrow elements within. Moreover, as mentioned above, it has been suggested in the literature that the identification of a specific subtype, namely nodular or dendriform, may help to narrow the differential diagnosis and etiology, but, more recently, it has also been proposed that either subtype may actually develop for a given etiology or that both subtypes can occur at the same time [11]. Therefore, the utility of lung biopsy after excluding potential underlying causes is somewhat debatable.

There is still no agreement on treatment and follow-up regimens in patients with DPO. Symptomatic treatment for cough or bronchial obstruction has been described, and imaging follow-up has also been suggested, although longitudinal data regarding the evolution of this condition are still limited [9].

In conclusion, DPO should be considered in different clinical-radiological settings since it occurs more frequently in the context of underlying interstitial lung diseases or extra-pulmonary disorders.

Multidisciplinary discussion is crucial for the final diagnosis.

## Informed consent

Informed consent was obtained from all individual participants included in the study.

## Contributors

All the authors contributed to the conception and design of the work and data collection. Serena Chiarello, Aldo Carnevale and Alberto Cossu analyzed the imaging data. Brunilda Marku cared for the patient and helped establish the diagnosis. Giovanni Lanza provided histological analysis.

All authors drafted the manuscript, critically revised the manuscript for important intellectual content, gave final approval of the version to be published and agreed to be accountable for all aspects of the work.

## Funding

The University of Ferrara supported this work by providing the publication fee.
